# Association of Functional Polymorphisms from Brain-Derived Neurotrophic Factor and Serotonin-Related Genes with Depressive Symptoms after a Medical Stressor in Older Adults

**DOI:** 10.1371/journal.pone.0120685

**Published:** 2015-03-17

**Authors:** Kerri S. Rawson, David Dixon, Petra Nowotny, William M. Ricci, Ellen F. Binder, Thomas L. Rodebaugh, Leah Wendleton, Peter Doré, Eric J. Lenze

**Affiliations:** 1 Department of Psychiatry, Washington University School of Medicine, Saint Louis, Missouri, United States of America; 2 Orthopaedic Trauma Service, Washington University School of Medicine / Barnes-Jewish Hospital, Saint Louis, Missouri, United States of America; 3 Department of Internal Medicine, Washington University School of Medicine, Saint Louis, Missouri, United States of America; 4 Department of Psychology, Washington University in Saint Louis, Saint Louis, Missouri, United States of America; Radboud University, NETHERLANDS

## Abstract

Depressive symptoms are common in older adults after a disabling medical event and interfere with rehabilitation and recovery from the disability. This prospective study examined the role of genetic polymorphisms implicated in synaptic integrity and stress-associated depression as predictors of depressive symptoms after hip fracture. We recruited healthy comparisons from the community and participants with hip fracture after surgical fixation from Saint Louis, Missouri hospitals. We examined the valine (Val) to methionine (Met) polymorphism in brain-derived neurotrophic factor (*BDNF*), serotonin 1A receptor (5HT1a-rs6295) polymorphism, and the serotonin transporter-linked polymorphic region (5HTTLPR) interaction with the rs25531 A to G single nucleotide polymorphism (5HTTLPR-rs25531) as predictors of depressive symptoms. We also examined whether depressive symptoms mediate the influence of *BDNF* genotype on functional recovery. Among 429 participants with hip fracture, *BDNF* Met/Met carriers developed significantly more depressive symptoms than Val/Val carriers during a four-week period after the fracture (p=.012). *BDNF* genotype also predicted functional recovery over the ensuing year, mediated by its effects on depressive symptoms (CI: 0.07-3.37). Unlike prior studies of stressful life events, the S′ 5HTTLPR-rs25531 variant did not predict higher levels of depressive symptoms; instead, we report an exploratory finding of an epistatic effect between *BDNF* and 5HTTLPR-rs25531 whereby the compounded effects of two LA alleles and *BDNF* Met/Met genotype elevate risk of depressive symptoms after hip fracture (p=.006). No differences between 5HT1a genotypes were found. Our findings suggest plasticity-related genetic factors contribute to the neural mechanisms of mental and functional well-being after a disabling medical stressor.

## Introduction

Disabling medical events, such as hip fracture, often trigger depressive symptoms. In fact, most of the total burden of depressive symptoms in our aging population occurs in the context of disabling medical events [1–3]. Hip fracture is a common [4] and severe life stressor [5,6]: it causes pain [7], fear [8], requires hospitalization and surgery [4,6], lengthy and intensive rehabilitation [9], and leads to increased risk of institutionalization and mortality [10–12]. Depressive symptoms after hip fracture have a pernicious effect on the recovery process: they are associated with higher discharge rates to and increased length of stay in nursing homes [13–15], poorer occupational and physical therapy participation, and poorer functional recovery [16,17]. Hip fracture patients with more depressive symptoms are less likely to return to pre-fracture levels in several areas of physical functioning (such as walking independently) as compared to patients with less depressive symptoms [16,18,19]. Currently, the ability to predict which persons are at-risk of depressive symptoms after a medical disability is surprisingly low. Identification of genetic risk factors has the potential to alert caregivers to monitor patients vulnerable for high depressive symptoms. Early-intervention and optimal management of depressive symptoms could then essentially mitigate their impact on functional recovery, thereby improving quality of life and functional independence in late-life.

The extent to which molecular processes contribute to the neurobiology of depressive symptoms and subsequent functional recovery after a medical stressor is not well understood. Past research indicates genes associated with synaptic integrity or those vulnerable to stressful events may influence depressive symptoms [20–22]. In this study, we investigate genetic variants previously associated with depressive symptoms by examining their impact on both depressive symptoms and functional recovery in older adults who have experienced a medical stressor.

### *BDNF* Val66Met polymorphism

Brain-derived neurotrophic factor (*BDNF*) is of the neurotrophin family [23] and has beneficial effects on health, particularly for promoting synaptic plasticity [20,24,25]. However, a valine (Val) to methionine (Met) substitution at nucleotide 66 (Val66Met; refSNP: rs6265) in the *BDNF* gene influences risk of depression in older adults [26–29]. In vivo experiments demonstrate the Met allele results in a reduction of activity-dependent BDNF secretion and a loss of BDNF protein at the synapse due to abnormal trafficking patterns [30,31]. In addition, the Met substitution is associated with reduced hippocampal activity [32] and atrophy of the hippocampus in humans [22,33,34], a structure important for mood regulation. The reduction in hippocampal volume likely reflects a decrease in BDNF protein at the synapse and, consequently, a decrease in synapse formation [30,35,36].

### Serotonin-related polymorphisms

In this study, we also assessed putative functional polymorphisms in serotonin (5HT), a hallmark neurotransmitter involved in depression. The gene *SLC6A4* encodes for the serotonin transporter (5HTT) and this transporter’s efficiency is altered by both a genetic variation of the serotonin transporter gene-linked polymorphic region (5HTTLPR) and a functional A to G single-nucleotide polymorphism (SNP; refSNP: rs25531) [37,38]. 5HTTLPR is a 44-bp repeat insertion (long) or deletion (short) in the promoter region resulting in different levels of serotonin transporter expression [39–41]. In depression research, the short (S) allele has been characterized as the susceptible allele due to its reduced ability to remove 5HT from the synaptic cleft compared to the long (L) allele [42,43]. Additional research has indicated the S and L alleles interact with the alleles of rs25531, such that the combination of the 5HTTLPR L allele and rs25531 G allele are equivalent to the S allele alone [38]. Although 5HTTLPR has been extensively studied in younger adults after stressful life events, few studies have examined it in late-life after a disabling medical event as the stressor. Older patients with myocardial infarction and at least one S allele had higher depressive scores than persons with two L alleles [40]. Similar results were found in older patients with a history of coronary disease [44], following a stroke [45], and in a small pilot study after hip fracture [46].

The *HTR1A* gene encodes the serotonin 1A receptor (5HT1a; refSNP: rs6295). 5HT1a hinders the release of serotonin and the G allele of C(-1019)G polymorphism is associated with increased depressive symptoms [47–50].

### Hypotheses

Here we examined functional polymorphisms from *BDNF* and serotonin-related genes as predictors of depressive symptoms after hip fracture. We hypothesized *BDNF* Met/Met genotype, 5HTTLPR-rs25531 S′ allele, and the G allele of the 5HT1a polymorphism would be associated with more depressive symptoms, and if so, they would also predict poorer functional recovery as a result of depressive symptoms. Lastly, we explored the interaction of *BDNF* and 5HTTLPR-rs25531 on depressive symptoms.

## Methods and Materials

### Setting and sample

We recruited participants with hip fracture from admissions to eight local hospitals in Saint Louis, Missouri from 2008–2012 and healthy comparisons from the community via self-referrals, presentations, advertisements, and the university’s research registry. Washington University's Human Research Protection Office, Washington University School of Medicine Institutional Review Board, and the review boards at the applicable participating research site (The Christian Hospital Research Committee for Christian Hospital, DePaul Institutional Review Board for DePaul Health Center, Missouri Baptist Medical Center Institutional Review Board for Missouri Baptist Medical Center, Saint Anthony’s Medical Center Institutional Review Board for Saint Anthony's Medical Center, SSM Saint Louis Institutional Review Board for SSM Saint Clare Health Center, Saint Louis University Institutional Review Board for Saint Louis University Medical Center Campus and Saint Mary’s Health Center, and Washington University School of Medicine Institutional Review Board for Barnes-Jewish Hospital) approved this study and approved the informed written consent document that was signed by all participants. Eligibility for participants with hip fracture included being age 60 or older, primary diagnosis of hip fracture to be surgically repaired, and absence of delirium or dementia. Participants with hip fracture were excluded if they were unable to provide written informed consent after complete description of the study, were unable to cooperate with the protocol, had a current diagnosis of major depression, had metastatic cancer, were significantly impaired by language, visual or hearing barriers, lived more than one hour away, were taking interferon medications, or had an inoperable fracture. Exclusion criteria for healthy comparisons were the same but also included persons who had a hip fracture in the past year, a major health issue, or had been hospitalized in the last six months.

The study was 52 weeks with evaluation time points at baseline (typically two days after surgery), week 1, 2, 4, 8, 12, 26, and 52. We conducted baseline, weeks 4 and 52 in person, and weeks 1, 2, 8, 12, and 26 by phone.

### Measures

Trained raters measured depressive symptoms with the Montgomery-Asberg Depression Rating Scale (MADRS) [51] at every time point. At baseline, MADRS scores are retrospective: participants described themselves for the week prior to hip fracture or entry to study (for healthy comparisons). Higher scores indicate more depressive symptoms. Major and Minor Depressive Disorder were assessed with the Structured Clinical Interview for Diagnostic and Statistical Manual of Medical Disorders-IV (SCID) [52] at baseline and any subsequent time point if MADRS > = 10 or if the depressed mood or anhedonia item was > = 2. Participants diagnosed with Major Depressive Disorder at baseline (reflecting current depression prior to hip fracture) were excluded. Test-retest reliability for MADRS was ICC = .84 (CI = 0.51–0.95), SCID past major depression κ = .52, and minor depression κ = .35.

Functional recovery was measured with the Functional Recovery Score (FRS) from the Hospital for Joint Diseases Geriatric Hip Fracture Research Group [53]. The FRS assesses Basic Activities of Daily Living, Instrumental Activities of Daily Living, and Mobility. A total score (0–100) is calculated from the three areas with higher scores indicating a greater ability to perform activities. Test-retest reliability was ICC = .75 (CI = 0.24–0.92).

Cognitive performance was assessed with the Short-Blessed Test [54] at baseline, week 4 and 52. Scores >10 warranted further evaluation to ensure absence of dementia or delirium.

Participants rated how stressful they have found their hip fracture as a measure of perceived stress. Ratings included: (1) not at all stressful, (2) somewhat stressful, or (3) very stressful [55]. Test-retest reliability was κ = .75.

### Genotyping

DNA was extracted from saliva and blood samples using standard procedures. For genotyping of the polymorphisms in the serotonin 1A receptor (rs6295), *BDNF* (rs6265), and 69 single nucleotide polymorphisms (SNPs) for the population stratification analysis, we used Sequenom technology, following manufacturer’s protocol. For serotonin transporter polymorphisms, we followed a protocol which genotypes the SLC6A4 promoter haplotype [38]. In short, it is a triplex polymerase chain reaction protocol followed by double restriction endonuclease digestion, determining the phase-certain 5HTTLPR VNTR and rs25531 genotypes which are combined as SA, SG, LA, and LG haplotypes. For analyses, a composite of 5HTTLPR-rs25531 was reflected by the number of LA alleles: LA/LA, LA/S′, and S′/S′ [38].

### Statistical Analysis

T-tests and chi-square were used to examine demographic and genotype differences between participants with hip fracture and healthy comparisons.

The main outcome, MADRS scores, had a skewed distribution. Thus, we used generalized estimating equations (GEE) with a Poisson distribution in SAS 9.3 procedure GENMOD (SAS Institute, Cary, NC) to examine the polymorphisms as predictors of depression symptoms. GEE is advised for instances where data will be correlated, such as repeated measurements, and for non-parametric data. GEE accounts for missed visits and dropouts using the all available pairs method. Within participants with hip fracture, we assessed main effects of each polymorphism for significance in separate models, with the most common homozygote as reference. We next ran separate GEE models to evaluate if age, gender, antidepressant use, education, or Short-Blessed Test were significant covariates. We then investigated the interaction between *BDNF* and 5HTTLPR-rs25531 to replicate prior findings. Significance level of p<.05 was set for main effects; however, due to testing for three polymorphisms, a probability value less than p<.0167 was considered significant.

We performed Kaplan-Meier Estimates of Survival Function to examine if the polymorphisms predicted Major and Minor Depressive Disorder. Due to low numbers of participants with hip fracture developing Major Depressive Disorder, we combined the two diagnoses. Additionally, we expanded the timeframe to eight weeks post-fracture to account for extra time needed for a clinical interview to make a SCID diagnosis after a MADRS score warranted further evaluation.

Lastly, mediation analysis using MEDIATE [56,57] in IBM SPSS Statistics 21.0 (Armonk, NY) was performed to examine *BDNF* effects on functional recovery through depressive symptoms. Using Helmert coding to address the multicategorical *BDNF* variable [57], estimation of coefficients for the first contrast compares Val/Val and Val/Met (Val+) relative to Met/Met while the second contrast compares Val/Val relative to Val/Met carriers. MADRS scores, averaged from weeks 1 through 4 to reflect high points of depressive symptoms post-fracture, served as the mediator. FRS was calculated as percent of baseline at week 12, 26, and 52. Indirect effects of *BDNF* on FRS at week 12, 26, and 52 were conducted using stratified bootstrap sampling based on 10,000 bootstrap samples. Significance of indirect effects was determined with biased-corrected confidence intervals (90%) not including zero [58]. Mediation analysis uses listwise deletion for participants missing data on variables included in each model.

We conducted population structure analysis with Structure Software Version 2.3 [59]. Thirty-five SNPs were selected from a panel of 69 SNPs that were in Hardy-Weinberg equilibrium. Structure parameters included the admixture model assuming independent allele frequencies with 10,000 burn-in period and 50,000 of MCMC reps. Project was run K 1–5 for five iterations. Results were imported into Structure Harvester Web v0.6.93 to detect the ideal number of clusters [60].

## Results

Our initial sample included 558 white (93%), 41 black (7%) and 3 Asian adults (<1%). Investigation of population stratification revealed two populations: those that self-identified as black and as white. Thus, to reduce variance explained by genetic diversity, data is presented for white participants only in the analyses [61,62]. At baseline (Table 1), participants with hip fracture were more likely to be female, to have less education, poorer functional scores, and worse Short-Blessed Test scores than healthy comparisons. We found no differences between participants with hip fracture and comparisons for age, MADRS depressive scores, or antidepressant use. Genotype frequencies from *BDNF-*rs6265, 5HTTLPR-rs25531, and 5HT1a-rs6295 were in Hardy-Weinberg equilibrium.

**Table 1:** Baseline demographic variables and summary of frequencies for ***BDNF*** Val66Met, 5HTTLPR-rs25531, and C(-1019)G 5HT1a (Caucasian only).

Characteristic	Healthy Comparisons (n = 92)		Participants with Hip Fracture (n = 466)			
	**Mean**	**SD**	**Mean**	**SD**	**t**	**p**
**Age (years)**	78.3	7.1	78.9	8.5	-0.73	.47
**Education, years** ^**a**^	15.2	2.8	13.04	2.8	6.45	<.001
**Short-Blessed Test**	1.9	2.5	4.6	3.3	-9.01	<.001
**MADRS Depression Score**	3.2	3.4	3.2	4.1	-0.19	.85
**Functional Recovery Score**	98.1	3.8	94.9	9.4	5.31	<.001
	**N**	**%**	**N**	**%**	**χ^2^**	**p**
**Female**	60	65	352	76	4.23	.04
**On Antidepressants** ^**a**^	15	17	97	21	0.66	.42
***BDNF* Val66Met** ^**a**^						
Val/Val	60	66	290	67		
Val/Met	30	33	123	29	0.03	.86
Met/Met	1	1	16	4		
**5HTTLPR-rs25531** ^**a**^						
S′/S′	22	24	110	25		
S′/LA	47	51	199	46	1.35	.51
LA/LA	23	25	125	29		
**5HT1a C(-1019)G** ^**a**^						
CC	27	30	95	22		
CG	44	48	229	54	2.36	.12
GG	20	22	100	24		

Abbreviations: 5HT1a, Serotonin 1A receptor; 5HTTLPR, serotonin transporter gene-linked polymorphic region; *BDNF*, brain-derived neurotrophic factor; MADRS, Montgomery-Asberg Depression Rating Scale.

^a^Reduced N

**Fig 1:**
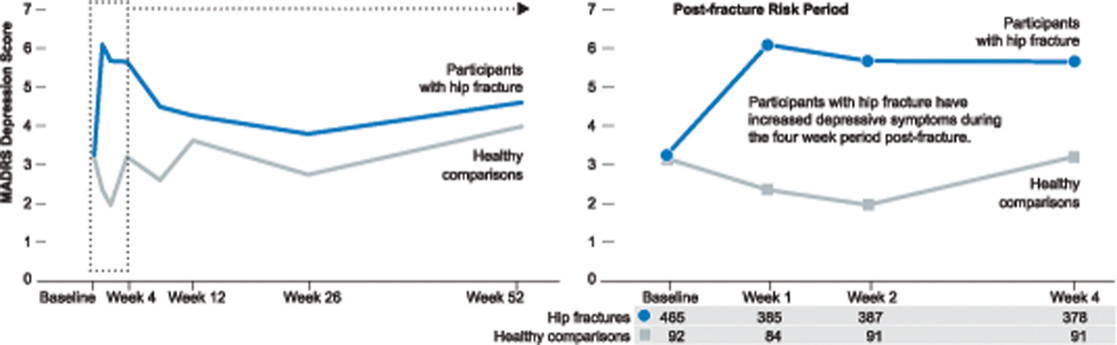
Observed Montgomery-Asberg Depression Rating Scale (MADRS) scores over time for participants with hip fracture and healthy comparisons. The number of participants with hip fracture and healthy comparisons are listed below the figure.

Our time course data shows hip fracture to be depressogenic from week 1 to 4, followed by a decline in depressive symptoms (Fig. 1). Therefore, to examine the candidate polymorphisms, we focused on the four-week period post-fracture where an increase in depressive scores among participants with hip fracture was observed relative to comparisons.

### Main genetic effects on depressive symptoms

**Fig 2:**
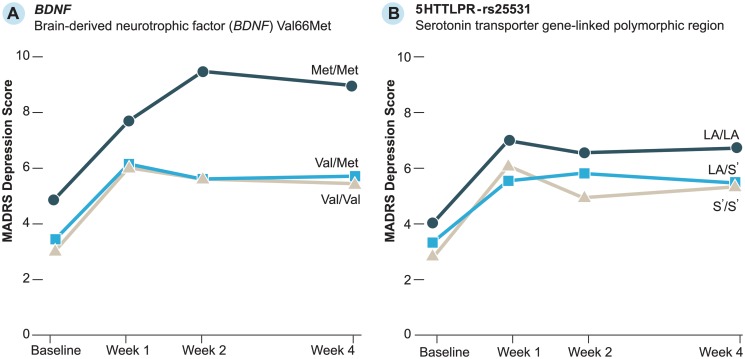
Observed Montgomery-Asberg Depression Rating Scale (MADRS) scores for participants with hip fracture.

Observed MADRS scores by *BDNF* and 5HTTLPR-rs25531 polymorphisms for participants with hip fracture are in Fig. 2. To examine differences in depression scores between genotypes, we entered *BDNF*, 5HTTLPR-rs25531, and C(-1019)G 5HT1a into separate GEE models that included time to account for repeated MADRS measures (Table 2). *BDNF* Met/Met carriers had significantly higher MADRS scores than Val/Val carriers (p = .012). For 5HTTLPR-rs25531, both S′/S′ (p = .032) and LA/S′ (p = .016) carriers had lower MADRS scores compared to LA/LA carriers. The 5HT1a model was not significant. Inclusion of all races/ethnicities in GEE models produced similar results (not shown).

**Table 2:** Parameter estimates (log) and empirical standard error estimates with time for four GEE models predicting MADRS depressive scores post-fracture.

Gene Predicting Depressive Scores		Estimate	SE	Z	p
***BDNF* Val66Met**	Intercept	1.72	0.05	31.77	<.001
(n = 429)	Time BL—Week 4	-0.58	0.07	-8.66	<.001
	Met/Met—Val/Val	0.39	0.16	2.50	.012
	Val/Met—Val/Val	0.01	0.09	0.16	.869
**5-HTTLPR/rs25531**	Intercept	1.88	0.07	26.54	<.001
(n = 434)	Time BL—Week 4	-0.58	0.07	-8.72	<.001
	S′/S′—LA/LA	-0.20	0.09	-2.15	.032
	LA/S′—LA/LA	-0.20	0.09	-2.40	.016
**5HT1a C(-1019)G**	Intercept	1.65	0.07	22.89	<.001
(n = 424)	Time BL—Week 4	-0.59	0.07	-8.84	<.001
	GG—CC	0.09	0.10	0.93	.353
	CG—CC	0.13	0.09	1.49	.136
***BDNF* Val66Met x**	Intercept	1.78	0.09	20.30	<.001
**5HTTLPR-rs25531**	Time BL—Week 4	-0.58	0.07	-8.64	<.001
(n = 428)	Met/Met—Val/Val	0.67	0.13	5.08	<.001
	Val/Met—Val/Val	0.22	0.13	1.65	.099
	S′/S′—LA/LA	-0.09	0.12	-0.72	.473
	LA/S′—LA/LA	-0.09	0.10	-0.89	.373
	Estimate 1^a^	-0.85	0.31	-2.76	.006^e^
	Estimate 2^b^	-0.21	0.31	-0.68	.494
	Estimate 3^c^	-0.24	0.20	-1.15	.250
	Estimate 4^d^	-0.32	0.20	-1.63	.104

Abbreviations: 5HT1a, Serotonin 1A receptor; 5HTTLPR, serotonin transporter gene-linked polymorphic region; *BDNF*, brain-derived neurotrophic factor; BL, baseline; GEE, generalized estimating equation; MADRS, Montgomery-Asberg Depression Rating Scale.

The estimated intercept (log) for each of the models refers to MADRS depressive scores for carriers of the reference (common homozygote) genotype. For example, irrespective of *BDNF* genotype, the intercept for all participants with hip fracture was 1.72 and relative to Val/Val carriers, Met/Met carriers had 0.39 units higher MADRS scores. The interaction estimates are interpreted as follows:

^a^The difference between Met/Met carriers and Val/Val carriers within LA/LA minus the difference between Met/Met carriers and Val/Val carriers within S′/S′.

^b^The difference between Met/Met carriers and Val/Val carriers within LA/LA minus the difference between Met/Met carriers and Val/Val carriers within LA/S′.

^c^The difference between Val/Met carriers and Val/Val carriers within LA/LA minus the difference between Val/Met carriers and Val/Val carriers within S′/S′.

^d^The difference between Val/Met carriers and Val/Val carriers within LA/LA minus the difference between Val/Met carriers and Val/Val carriers within LA/S′.

^e^Contrast results for GEE analysis of the interaction indicate a significant difference between *BDNF* Met/Met and Val/Val carriers within LA/LA (*χ* ^2^ = 4.37(1), p = .037) and between *BDNF* Met/Met and Val/Met carriers within LA/LA (*χ* ^2^ = 3.86(1), p = .050).

We next examined which covariates were predictive of MADRS scores. Gender, age, education, and Short-Blessed Test scores were not significant. Antidepressant use significantly predicted MADRS scores (b = 0.24, SE = .08, p = .005). The polymorphisms were therefore re-examined after including time and antidepressant use (S1 Table). Antidepressant use remained significant in all models, indicating participants on antidepressants had higher MADRS scores. Like the model without antidepressant use, the same trends were seen for *BDNF*, 5HTTLPR-rs25531, and 5HT1a.

### Exploring an interaction between *BDNF* and 5HTTLPR-rs25531

**Fig 3:**
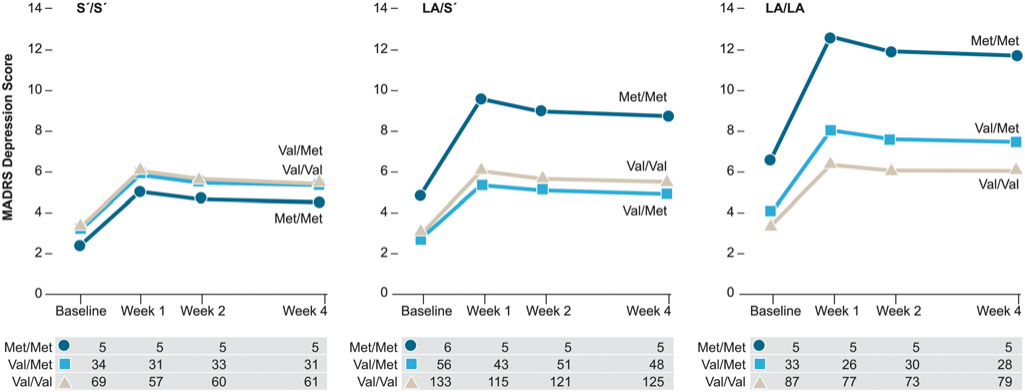
Predicted values for the interaction effect between brain-derived neurotrophic factor (*BDNF*) and serotonin transporter gene-linked polymorphic region, 5HTTLPR-rs25531 . Within hip fracture participants with two 5HTTLPR-rs25531 LA alleles (LA/LA), contrast results for the GEE analysis indicate *BDNF* Met/Met carriers had significantly higher depressive symptoms than Val/Val carriers (χ^2^ = 4.37(1), p = .037) and Val/Met carriers (χ^2^ = 3.86(1), p = .05). Sample sizes are listed below each graph. Abbreviations: MADRS, Montgomery-Asberg Depression Rating Scale; GEE, Generalized estimating equations.

Table 2 also summarizes the significant interaction between *BDNF* and 5HTTLPR-rs25531. As displayed in Fig. 3, Met/Met carriers had significantly higher MADRS scores than Val/Val carriers among participants with two LA alleles, whereas this distinction was not observed among S′ carriers. The interaction remained significant after including time and antidepressant use (p = .014; S1 Table).

### Genetic results for participants with high-perceived stress

We further analyzed results by including only participants who perceived the hip fracture as stressful. Five percent of participants who rated the hip fracture as “not at all stressful” had lower MADRS scores than 68% of participants who rated it “very stressful” (p = .005) and 27% of participants who rated it “somewhat stressful” (p<.001). Analyses were re-examined by excluding those who reported the event as being “not at all stressful” (S2 Table). As in the aforementioned results, we found the same trends for the main effects of the at-risk polymorphisms using uncorrected p-values in this high-perceived stressed subsample. The interaction between *BDNF* and 5HTTLPR-rs25531 remained significant (p = .011).

### Genetic risk for Major and Minor Depressive Disorder

We examined if the polymorphisms predicted incident depression diagnosis during the eight weeks post-fracture. The log rank tests from Kaplan-Meier survival estimates indicated no significant differences between genotypes for *BDNF* Val66Met (χ^2^ = 0.81(2), p = .67), 5HTTLPR-rs25531 (χ^2^ = 0.31(2), p = .86), or 5HT1a (χ^2^ = 2.36(2), p = .31).

### Mediation test of *BDNF* on FRS through depressive symptoms

**Fig 4:**
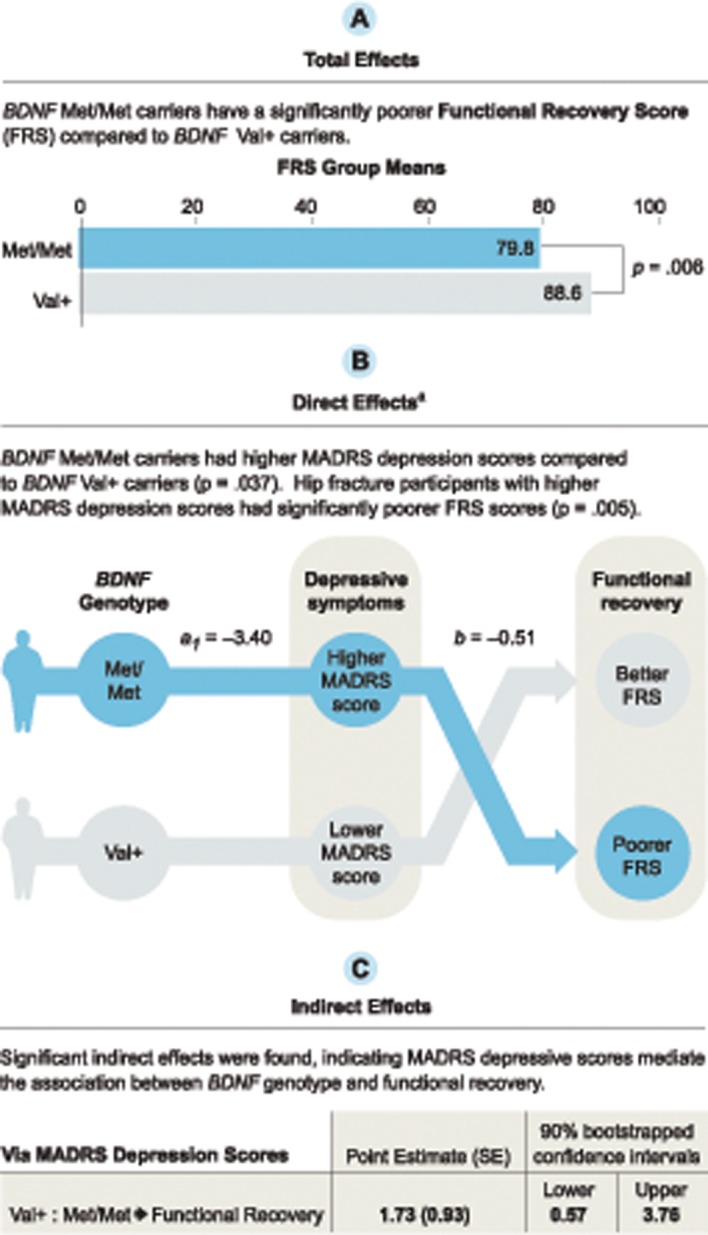
Mediation model using Hayes’ (2013) multicategorical independent variable method . Mediation test of the relationship between brain-derived neurotrophic factor (*BDNF*) Val66Met polymorphism and the Functional Recovery Score (FRS) at week 12 as a result of the Montgomery-Asberg Depression Rating Scale (MADRS) depression scores. Results are shown for the *BDNF* Val+:Met/Met contrast. The Val/Val:Val/Met contrast was not significant.

^a^Independent of MADRS scores, FRS was not influenced by the direct effect of *BDNF* Val+ relative to Met/Met (p = .09).

The indirect effects from the mediation analysis were significant and indicated *BDNF* genotype affects functional recovery as a result of depressive symptoms at weeks 12, 26, and 52 (S3 Table). Total, direct, and indirect effects for the Val+:Met/Met contrast at week 12 are provided in Fig. 4 for illustration. The total effect indicated Val+ carriers had significantly better FRS than Met/Met carriers at week 12. The direct effects indicated Val+ carriers had significantly lower MADRS scores relative to Met/Met carriers and participants with lower MADRS scores had higher FRS, regardless of *BDNF* genotype, at all 3 time points. Indirect effects indicated MADRS scores are a significant mediator for the Val+:Met/Met contrast. That is, Val+ carriers scored an average 1.728 units higher on FRS at week 12 relative to Met/Met carriers due to the effect of *BDNF* genotype on depressive symptoms. Similar results were seen using FRS as the outcome at week 26 and 52. Neither total, direct, nor indirect effects for the Val/Val:Val/Met contrast were significant. There was no evidence that *BDNF* genotype influenced FRS independent of its effect on depressive symptoms at week 12, 26, or 52 for either contrast.

## Discussion

Depressive symptoms are commonplace after a disabling medical event, can increase risk and length of stay in nursing homes, and impede functional recovery [13,16,17]. In this study, we sought to identify responsible genetic mechanisms underlying the neurobiological mental and functional consequences that arise after a medical stressor.

This longitudinal analysis provided several noteworthy findings. Results indicate hip fracture participants with two *BDNF* Met alleles have higher depressive symptoms than Val/Val carriers in the first weeks post-fracture. Analyses also indicated *BDNF* Met/Met carriers were more likely to have poorer functional recovery scores for a year after hip fracture as a result of *BDNF* genotype on depressive symptoms. We did not find the expected association between the 5HTTLPR-rs25531 short allele and depressive symptoms; however, we report an interaction between *BDNF* and 5HTTLPR-rs25531. Although the interaction needs to be further explored in a more suitably sized cohort, our current sample indicated *BDNF* Met/Met carriers have higher depressive symptoms than Val/Met and Val/Val carriers in the presence of two LA alleles.

Understanding the neurobiological consequences of genes arising after a medical stressor is not only important for identifying older adults at risk of higher depressive symptoms, but secondarily, as our data (and others’) suggests [63,64], also significant for their functional recovery. *BDNF* generally plays an important role in synaptic plasticity [20], however, the Met allele at the Val66Met polymorphism results in abnormal neurotrophin trafficking patterns in neuronal cells. More specifically, the BDNF Val protein is localized more in the axon terminal, whereas expression of the BDNF Met protein has most localization in the cell body [30,31]. In consequence, a loss of BDNF at the axonal terminal among Met/Met carriers may compromise formation of new synapses [30,31,33,35,36]. Considering previous studies have indicated that atrophy of the hippocampus [22,33] and reduced hippocampal activity [32] are associated with the Met allele, *BDNF* Met/Met carriers may be vulnerable to the development of depressive symptoms due to inadequate synaptic formation in the hippocampus, a structure important for mood regulation [26,34,35,65,66].

The serotonergic system has long been a focus of research on the genetic origins of depression. Studies, like Caspi’s [42], investigating 5HTTLPR and depression, have been particularly arguable in the context of gene-environment effects [67,68]. While several previous studies have observed the 5HTTLPR short allele is related to depression due to less efficient transcription of serotonin transporters [43], others have found no association of 5HTTLPR and depression [69]. The different conclusions may be due to prior observations having smaller sample sizes [46], examining a history of life events rather than an acute medical event, having younger subjects, or not accounting for the influence of rs25531 A>G on 5HTTLPR [70]. Our current observations suggest the phenomenon may be pathophysiologically different after a medical stressor, or as Taylor and colleagues [71] findings indicate, neurostructural differences are evident between early and late-onset depression. Among older adults classified as having depression after age 50, results showed those who were homozygous with the L/L genotype had smaller hippocampal volume than those with early-onset depression. Future efforts should include the concerted genetic mechanisms among serotonin-related polymorphisms as well as compare genetic differences between persons with progressive medical events (i.e. stroke, heart disease) to those with injury-related medical events as investigated here.

We also explored a gene-gene interaction due to existing reports linking neurotrophins with serotonin in depression literature [72,73] and research suggesting compounded effects of certain genes can heighten risk of psychiatric symptoms [74]. In this study, the interaction between *BDNF* and 5HTTLPR-rs25531 was specific to LA/LA carriers. Particularly, significant differences in depressive symptoms were found between Met/Met and Val+ carriers among LA/LA carriers only; the interaction was not significant among S′ carriers. Thus, the short allele appears to contribute protection against depressive symptoms in persons with the at-risk *BDNF* genotype. While an inclusive model explaining the neurotrophin-serotonin association is unknown, past research indicates a dynamic relationship between the two signaling systems. The BDNF protein contributes to the survival and plasticity of serotonergic neurons as well as plays a role in serotonin secretion via BDNF’s TrkB receptors. Additional studies have also demonstrated stress reduces BDNF and serotonin levels [72,73]. Conceivably, and like most bodily systems, a homeostatic equilibrium would be achieved between the two signaling systems under normal conditions regardless of one’s *BDNF* and 5HTTLPR-rs25531 alleles. However, it could be speculated that LA/LA carriers with normal functioning 5HT transcription, combined with the suboptimal *BDNF* Met substitution, are more vulnerable during a stressful situation. That is, a further reduction of BDNF due to stress could impact BDNF’s ability to contribute to the survival and functioning of serotoninergic neurons, whereas the less functional serotonin system among S′ carriers has learned to adapt with neurotrophins over time and may be more resistant to stressors. Further studies, specifically ones that reveal how the neurotrophin-serotonin systems adapt to maintain homeostasis over time and how they interact as a result of a stressful experience at the biological level, are needed.

Similar results have also been found for neuroticism and cortisol levels: *BDNF* Met carriers homozygous for 5HTTLPR L allele have increased neuroticism scores [75], and, in children, *BDNF* Met carriers with at least one 5HTTLPR L allele have higher cortisol levels [76]. In contrast, studies investigating stressful life events found higher depression among Korean older adults [28] and maltreated children [77] with *BDNF* Met and 5HTTLPR S′ allele. As opposed to limiting investigations to a single gene, further studies examining net effects of *BDNF* and serotonin-related polymorphisms may better explain stress-associated depression risk factors [62]. This is particularly true in consideration of the complexity underlying depression pathophysiology.

In contrast to the high levels of depressive symptoms in this sample, a low number of participants with hip fracture received a clinical diagnosis of depression. Likely, due to these low rates, we did not find that our candidate polymorphisms were related to a clinical diagnosis. However, depressive symptoms, whether or not they meet criteria for a clinical diagnosis of depression, are still dangerous for patient’s health and are a barrier to successful recovery.

This was a study specifically designed to test for plasticity-related genetic effects on depressive symptoms after a disabling medical stressor. Depression is a heterogeneous disease, but in this project we utilized hip fracture as a sort of natural experiment to test candidate genetic factors explicitly. This study included immediate recruitment of participants after hip fracture and careful characterization of depressive symptoms.

Limitations included a small number of *BDNF* Met/Met carriers. The interaction finding is preliminary and requires replication. A replication study could be accomplished in several ways including studying BDNF levels, downstream functioning, or a sample with a higher number of *BDNF* Met/Met carriers. Prior studies suggest the Met allele is more common in certain Asian populations [78] which may be a more ideal population to further examine Met/Met effects.

In conclusion, our data indicates plasticity-related polymorphisms are associated with higher depressive symptoms after a medical stressor in late-life and, subsequently, can negatively affect recovery. This knowledge is crucial for our aging population’s need to maintain independence. In addition, our data suggests a neurotrophin-serotonin link. As Kendler [79] argues, it is unlikely we will uncover an exclusive genetic pattern revealing a heterogeneous illness such as depression; but it is possible to identify subsets of genetic interactions that delineate particular molecular pathways susceptible under certain genetic and environmental conditions. The interaction identified here highlights the need to further study and understand the neurobiological outcomes resulting from neurotrophin and neurotransmitter genetic interactions in stress-associated depression.

## Supporting Information

S1 Table Parameter estimates (log) and empirical standard error estimates with time and antidepressant use entered as covariates for four GEE models predicting MADRS depressive scores post-fracture. Abbreviations: 5HT1a, Serotonin 1A receptor; 5-HTTLPR, serotonin transporter gene-linked polymorphic region; *BDNF*, brain-derived neurotrophic factor; BL, baseline; GEE, generalized estimating equation; MADRS, Montgomery-Asberg Depression Rating Scale.
(DOCX)

S2 Table Parameter estimates (log) and empirical standard error estimates in the high-perceived stress subsample post-fracture (excluding five percent of participants who rated the hip fracture as “not at all stressful”) with time and antidepressant use entered as covariates. Four separate GEE models predicting MADRS depressive scores post-fracture. Abbreviations: 5HT1a, Serotonin 1A receptor; 5HTTLPR, serotonin transporter gene-linked polymorphic region; *BDNF*, brain-derived neurotrophic factor; BL, baseline; GEE, generalized estimating equation; MADRS, Montgomery-Asberg Depression Rating Scale.
(DOCX)

S3 Table Mediation analysis examining the effect of the *BDNF* Val66Met polymorphism on Functional Recovery Score (FRS) as a result of MADRS Depressive Scores. Abbreviations: *BDNF*, brain-derived neurotrophic factor; MADRS, Montgomery-Asberg Depression Rating Scale; FRS, Functional Recovery Score.
(DOCX)

S1 File Supporting Information eReferences. (DOCX)
