# Correction: Association of Functional Polymorphisms from Brain-Derived Neurotrophic Factor and Serotonin-Related Genes with Depressive Symptoms after a Medical Stressor in Older Adults

**DOI:** 10.1371/journal.pone.0126451

**Published:** 2015-04-17

**Authors:** The PLOS ONE Staff

Fig 1 and Fig 4 appear at a low resolution. Please see the corrected Fig 1 and Fig 4 here. The publisher apologizes for these errors.

**Fig 1:**
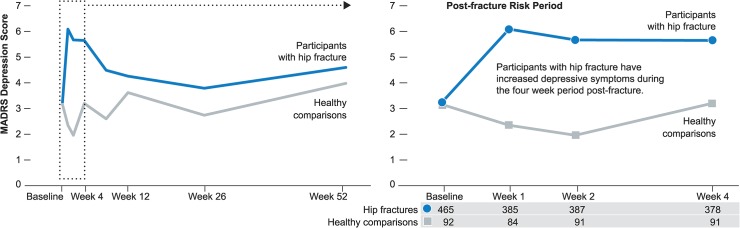
Observed Montgomery-Asberg Depression Rating Scale (MADRS) scores over time for participants with hip fracture and healthy comparisons. The number of participants with hip fracture and healthy comparisons are listed below the figure.

**Fig 4:**
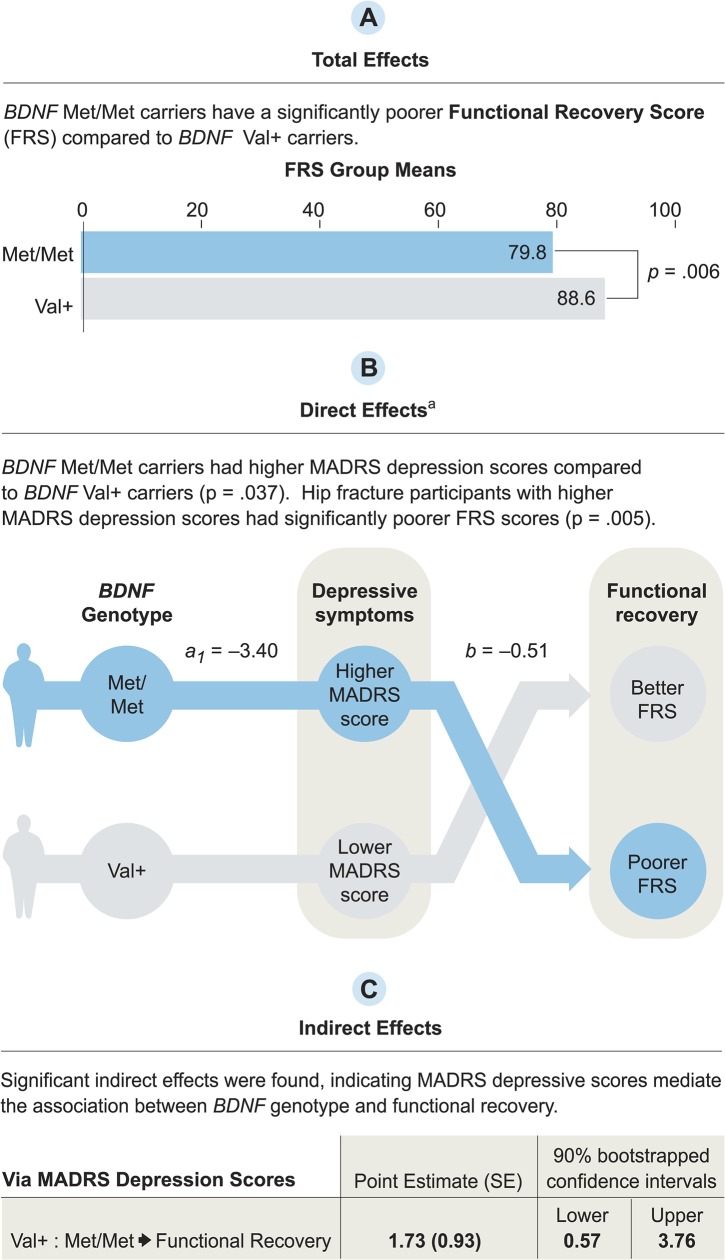
Mediation model using Hayes’ (2013) multicategorical independent variable method. Mediation test of the relationship between brain-derived neurotrophic factor (*BDNF*) Val66Met polymorphism and the Functional Recovery Score (FRS) at week 12 as a result of the Montgomery-Asberg Depression Rating Scale (MADRS) depression scores. Results are shown for the *BDNF*Val+:Met/Met contrast. The Val/Val:Val/Met contrast was not significant.
