# Supplementary material for: Association of Functional Polymorphisms from Brain-Derived Neurotrophic Factor and Serotonin-Related Genes with Depressive Symptoms after a Medical Stressor in Older Adults
Source: PLoS One. 2015 Mar 17;10(3):e0120685. doi: 10.1371/journal.pone.0120685 (PMC4363147; doi:10.1371/journal.pone.0120685)
Supplement: S2 Table — Four separate GEE models predicting MADRS depressive scores post-fracture. Abbreviations: 5HT1a, Serotonin 1A receptor; 5HTTLPR, serotonin transporter gene-linked polymorphic region; BDNF, brain-derived neurotrophic factor; BL, baseline; GEE, generalized estimating equation; MADRS, Montgomery-Asberg Depression Rating Scale. (DOCX) [file pone.0120685.s002.docx]

**S2 Table. Parameter estimates (log) and empirical standard error estimates in the high-perceived stress subsample post-fracture (excluding five percent of participants who rated the hip fracture as “not at all stressful”) with time and antidepressant use entered as covariates. Four GEE models predicting MADRS depressive scores post-fracture.**

| Gene Predicting Depressive Symptoms | | Estimate | SE | Z | p |
| --- | --- | --- | --- | --- | --- |
| *BDNF* Val66Met | Intercept | 1.69 | 0.06 | 29.75 | <.001 |
| (n = 400) | Time BL – Week 4 | -0.58 | 0.07 | -8.53 | <.001 |
|  | Antidepressant use | 0.26 | 0.09 | 3.04 | .002 |
|  | Met/Met – Val/Val | 0.36 | 0.16 | 2.29 | .022 |
|  | Val/Met – Val/Val | 0.02 | 0.09 | 0.28 | .776 |
| 5HTTLPR-rs25531 | Intercept | 1.84 | 0.07 | 24.90 | <.001 |
| (n = 405) | Time BL – Week 4 | -0.58 | 0.07 | -8.57 | <.001 |
|  | Antidepressant use | 0.26 | 0.09 | 2.98 | .003 |
|  | S′/S′ – LA/LA | -0.20 | 0.10 | -2.12 | .034 |
|  | LA/S′ – LA/LA | -0.19 | 0.09 | -2.17 | .030 |
| 5HT1a C(-1019)G | Intercept | 1.60 | 0.07 | 21.37 | <.001 |
| (n = 395) | Time BL – Week 4 | -0.59 | 0.07 | -8.69 | <.001 |
|  | Antidepressant use | 0.27 | 0.09 | 3.07 | .002 |
|  | GG – CC | 0.13 | 0.10 | 1.35 | .176 |
|  | CG – CC | 0.14 | 0.09 | 1.67 | .095 |
| *BDNF* Val66Met x | Intercept | 1.74 | 0.09 | 18.99 | <.001 |
| 5HTTLPR-rs25531 | Time BL – Week 4 | -0.58 | 0.07 | -8.49 | <.001 |
| (n = 399) | Antidepressant use | 0.25 | 0.09 | 2.92 | .004 |
|  | Met/Met – Val/Val | 0.60 | 0.14 | 4.47 | <.001 |
|  | Val/Met – Val/Val | 0.21 | 0.13 | 1.60 | .110 |
|  | S′/S′ – LA/LA | -0.08 | 0.12 | -0.69 | .489 |
|  | LA/S′ – LA/LA | -0.08 | 0.11 | -0.77 | .44 |
|  | Estimate 1^a^ | -0.80 | 0.31 | -2.55 | .011^e^ |
|  | Estimate 2^b^ | -0.16 | 0.32 | -0.50 | .617 |
|  | Estimate 3^c^ | -0.24 | 0.21 | -1.13 | .258 |
|  | Estimate 4^d^ | -0.29 | 0.20 | -1.46 | .146 |

Abbreviations: 5HT1a, Serotonin 1A receptor; 5HTTLPR, serotonin transporter gene-linked polymorphic region; *BDNF,* brain-derived neurotrophic factor; BL, baseline; GEE, generalized estimating equation; MADRS, Montgomery-Asberg Depression Rating Scale.

The estimated intercept (log) for each of the models refers to MADRS depressive scores for persons not on antidepressants and carriers of the reference (common homozygote) genotype. For example, irrespective of *BDNF* genotype, the intercept for all participants with hip fracture was 1.69 and relative to Val/Val carriers, Met/Met carriers had 0.36 units higher MADRS scores.

The interaction estimates are interpreted as follows:

^a^The difference between Met/Met carriers and Val/Val carriers within LA/LA minus the difference between Met/Met carriers and Val/Val carriers within S′/S′.

^b^The difference between Met/Met carriers and Val/Val carriers within LA/LA minus the difference between Met/Met carriers and Val/Val carriers within LA/S′

^c^The difference between Val/Met carriers and Val/Val carriers within LA/LA minus the difference between Val/Met carriers and Val/Val carriers within S′/S′.

^d^The difference between Val/Met carriers and Val/Val carriers within LA/LA minus the difference between Val/Met carriers and Val/Val carriers within LA/S′.

^e^Contrast results for GEE analysis of the interaction indicate a significant difference between Met/Met and Val/Val carriers within LA/LA only (χ^2^ = 4.32(1), *P* = .038).
