# Supplementary material for: Association of Functional Polymorphisms from Brain-Derived Neurotrophic Factor and Serotonin-Related Genes with Depressive Symptoms after a Medical Stressor in Older Adults
Source: PLoS One. 2015 Mar 17;10(3):e0120685. doi: 10.1371/journal.pone.0120685 (PMC4363147; doi:10.1371/journal.pone.0120685)
Supplement: S3 Table — Abbreviations: BDNF, brain-derived neurotrophic factor; MADRS, Montgomery-Asberg Depression Rating Scale; FRS, Functional Recovery Score. (DOCX) [file pone.0120685.s003.docx]

**S3 Table. Mediation analysis examining the effect of the *BDNF* Val66Met polymorphism on Functional Recovery Score (FRS) as a result of MADRS Depressive Scores.**

|  | **Mediator** | | | | **Total Effect** | | | | **Direct Effects** | | | | | | | | **Indirect Effect** | | | | | | | | | |
| --- | --- | --- | --- | --- | --- | --- | --- | --- | --- | --- | --- | --- | --- | --- | --- | --- | --- | --- | --- | --- | --- | --- | --- | --- | --- | --- |
|  | ***MADRS Score*** | | | | ***Functional Recovery Score*** | | | | ***Functional Recovery Score*** | | | | | | | | *BDNF ⯈ MADRS ⯈ FRS* | | | | | | | | | |
| **Week 12** |  | **Est** | **SE** | **p** |  | **Est** | **SE** | **p** |  | | **Est** | | **SE** | | **p** | |  | | **Est** | | **SE** | | **LCI** | | **UCI** | |
| Intercept | *i_1_* | 6.76 | 0.44 | <.001 | *i_3_* | 85.69 | 1.51 | <.001 | *i_2_* | | 89.13 | | 1.92 | | <.001 | |  | |  | |  | |  | |  | |
| Val+ : Met/Met | *a_1_* | -3.40 | 1.24 | .006 | *c_1_* | 8.82 | 4.22 | .037 | *c'_1_* | | 7.10 | | 4.22 | | .094 | | *a_1_b* | | 1.73 | | 0.93 | | 0.57 | | 3.76 | |
| Val/Val : Val/Met | *a_2_* | 0.09 | 0.54 | .869 | *c_2_* | -0.09 | 1.85 | .963 | *c'_2_* | | -0.04 | | 1.84 | | .983 | | *a_2_b* | | -0.05 | | 0.29 | | -0.57 | | 0.39 | |
| MADRS Scores | -- | -- | -- | -- | -- | -- | -- | -- | *b* | | -0.51 | | 0.18 | | .005 | |  | |  | |  | |  | |  | |
|  | *R^2^* = 0.02 | | | | *R^2^* = 0.01 | | | | *R^2^* = 0.03 | | | | | | | |  | | | | | | | | | |
|  | *F*(2, 354) = 3.79, *P* = .02 | | | | *F*(2, 354) = 2.20, *P* = .11 | | | | *F*(3, 353) = 4.16, *P* = .007 | | | | | | | |  | | | | | | | | | |
| **Week 26** |  | **Est** | **SE** | **p** |  | **Est** | **SE** | **p** |  | | **Est** | | **SE** | | **p** | |  | | **Est** | | **SE** | | **LCI** | | **UCI** | |
| Intercept | *i_1_* | 6.54 | 0.48 | <.001 | *i_3_* | 91.94 | 1.41 | <.001 | *i_2_* | | 95.41 | | 1.74 | | <.001 | |  | |  | |  | |  | |  | |
| Val+ : Met/Met | *a_1_* | -3.23 | 1.35 | .017 | *c_1_* | 4.68 | 3.97 | .239 | *c'_1_* | | 2.96 | | 3.94 | | .453 | | *a_1_b* | | 1.72 | | 1.18 | | 0.35 | | 4.44 | |
| Val/Val : Val/Met | *a_2_* | 0.32 | 0.57 | .576 | *c_2_* | -1.84 | 1.68 | .275 | *c'_2_* | | -1.67 | | 1.65 | | .315 | | *a_2_b* | | -0.17 | | 0.35 | | -0.91 | | 0.23 | |
| MADRS Scores | -- | -- | -- | -- | -- | -- | -- | -- | *b* | | -0.53 | | 0.16 | | .001 | |  | |  | |  | |  | |  | |
|  | *R^2^* = 0.02 | | | | *R^2^* = 0.007 | | | | *R^2^* = 0.04 | | | | | | | |  | | | | | | | | | |
|  | *F*(2, 331) = 2.94, *P* = .05 | | | | *F*(2, 331) = 1.18, *P* = .31 | | | | *F*(3, 330) = 4.50, *P* = .004 | | | | | | | |  | | | | | | | | | |
| **Week 52** |  | **Est** | **SE** | **p** |  | **Est** | **SE** | **p** |  | **Est** | | **SE** | | **p** | |  | | **Est** | | **SE** | | **LCI** | | **UCI** | |  |
| Intercept | *i_1_* | 6.50 | 0.45 | <.001 | *i_3_* | 91.02 | 1.36 | <.001 | *i_2_* | 93.13 | | 1.73 | | <.001 | |  | |  | |  | |  | |  | |  |
| Val+ : Met/Met | *a_1_* | -3.22 | 1.27 | .012 | *c_1_* | 5.82 | 3.81 | .127 | *c'_1_* | 4.77 | | 3.83 | | .21 | | *a_1_b* | | 1.05 | | 0.92 | | 0.07 | | 3.37 | |  |
| Val/Val : Val/Met | *a_2_* | 0.22 | 0.56 | .691 | *c_2_* | -2.25 | 1.69 | .184 | *c'_2_* | -2.18 | | 1.68 | | .20 | | *a_2_b* | | -0.07 | | 0.23 | | -0.62 | | 0.16 | |  |
| MADRS Scores | -- | -- | -- | -- | -- | -- | -- | -- | *b* | -0.33 | | 0.17 | | .05 | |  | |  | |  | |  | |  | |  |
|  | *R^2^* = 0.02 | | | | *R^2^* = 0.01 | | | | *R^2^* = 0.02 | | | | | | |  | | | | | | | | | |  |
|  | *F*(2, 319) = 3.22, *P* = .04 | | | | *F*(2, 319) = 1.88, *P* = .15 | | | | *F*(3, 318) = 2.53, *P* = .06 | | | | | | |  | | | | | | | | | |  |

Abbreviations: *BDNF*, brain-derived neurotrophic factor; MADRS, Montgomery-Asberg Depression Rating Scale; FRS, Functional Recovery Score

*Notes:*  Mediation model coefficients reported from SPSS 21 Mediate using Hayes’ (2013) Multicategorical Independent Variable Method^1^*.* Helmert coding was used to dummy code the contrasts. The first contrast estimates reflect *BDNF* Val/Met and Val/Val (Val+) relative to Met/Met. The second contrast estimates reflect *BDNF* Val/Val relative to Val/Met. MADRS Depressive Scores were averaged over weeks 1 through 4 to reflect the highest levels of depressive symptoms post-fracture. Higher scores are indicative of more depressive symptoms. Functional Recovery Scores (FRS) were calculated as percent of baseline at week 12, week 26, and week 52 for mediation analysis. Lower scores are indicative of poorer functional recovery. Inference of indirect effects determined by bias-corrected bootstrap 90% confidence intervals based on 10,000 bootstrap samples not including zero.
