# Supplementary material for: Association of Functional Polymorphisms from Brain-Derived Neurotrophic Factor and Serotonin-Related Genes with Depressive Symptoms after a Medical Stressor in Older Adults
Source: PLoS One. 2015 Mar 17;10(3):e0120685. doi: 10.1371/journal.pone.0120685 (PMC4363147; doi:10.1371/journal.pone.0120685)
Supplement: S1 File — (DOCX) [file pone.0120685.s004.docx]

**S1 File. Supplementary Information eReferences.**

1. Hayes AF, Preacher KJ. Statistical mediation analysis with a multicategorical independent variable. *Br J Math Stat Psychol*. 2013. doi:10.1111/bmsp.12028.
